# Targeting Arthrogenic Muscle Inhibition in Chronic Ankle Instability: A Narrative Review of Neural and Functional Rehabilitation Strategies

**DOI:** 10.3390/medicina61071267

**Published:** 2025-07-13

**Authors:** Roberto Tedeschi, Federica Giorgi, Danilo Donati

**Affiliations:** 1 Independent Researcher, 40100 Bologna, Italy; 2 Pediatric Physical Medicine and Rehabilitation Unit, IRCCS Institute of Neurological Sciences, 40139 Bologna, Italy; federica.giorgi15@gmail.com; 3 Physical Therapy and Rehabilitation Unit, Policlinico di Modena, 41125 Modena, Italy; danilo.donati@unimore.it; 4 Clinical and Experimental Medicine PhD Program, University of Modena and Reggio Emilia, 41121 Modena, Italy

**Keywords:** chronic ankle instability, arthrogenic muscle inhibition, neuromodulation, tDCS, rehabilitation

## Abstract

*Background and Objectives*: Arthrogenic muscle inhibition (AMI) is a key neurophysiological barrier to effective rehabilitation in individuals with chronic ankle instability (CAI). The primary objective of this narrative review is to explore the role of arthrogenic muscle inhibition (AMI) in chronic ankle instability (CAI) and to critically appraise neurophysiological and rehabilitative strategies targeting its resolution. Although peripheral strengthening remains a cornerstone of treatment, the roles of spinal and cortical modulation are increasingly recognised. *Materials and Methods:* A narrative review was conducted to examine recent clinical trials targeting AMI in CAI populations. A structured search of MEDLINE, Web of Science, Scopus, Cochrane Central, and PEDro was performed. Five studies were included, encompassing peripheral, spinal, and cortical interventions. The outcomes were grouped and analysed according to neurophysiological and functional domains. *Results:* Manual therapy combined with exercise improved pain, strength, and functional mobility. Fibular reposition taping transiently enhanced spinal reflex excitability, while transcranial direct current stimulation (tDCS) over the primary motor cortex significantly modulated corticospinal excitability and voluntary muscle activation. Improvements in subjective stability, dynamic balance, and neuromuscular responsiveness were observed in the majority of the five included studies, although methodological heterogeneity and short-term follow-ups limit generalisability. *Conclusions:* Multimodal interventions targeting different levels of the neuromotor system appear to be more effective than isolated approaches. Integrating manual therapy, sensorimotor training, and neuromodulation may optimise outcomes in CAI rehabilitation. Future trials should focus on standardised outcome measures and long-term efficacy.

## 1. Introduction

Arthrogenic muscle inhibition (AMI) is an involuntary neural response that restricts the full activation of a muscle surrounding a joint following injury, inflammation, or degeneration [1]. Although originally recognised and extensively studied in relation to the quadriceps muscle after knee trauma, accumulating evidence indicates that AMI also affects other joints such as the ankle, hip, and shoulder [2]. For example, McVey et al. [3] reported reduced soleus H-reflex amplitudes following lateral ankle sprain, suggesting spinal-level inhibition; Kim et al. [4] observed AMI-related deficits in hip abductors after acetabular labral tears; and Ingersoll et al. [5] described persistent supraspinal inhibition following shoulder injury. These findings reinforce the notion that AMI is a generalised neurophysiological response, not limited to the knee. It may also significantly affect other peripheral joints, including the ankle, where its role has often been underestimated or overlooked [5]. AMI is not caused by structural damage to the muscle itself, but by a series of neurophysiological alterations in the joint environment, which affect afferent sensory input and spinal reflex excitability, ultimately limiting motor unit recruitment [6]. This inhibitory phenomenon is thought to be protective in origin, aiming to reduce further joint damage, yet its persistence beyond the acute phase of injury may compromise functional recovery and contribute to chronic joint instability [7].

In the case of the ankle, the implications of AMI are particularly relevant due to the high prevalence of lateral ankle sprains and their transition into chronic ankle instability (CAI). Epidemiological studies report that approximately 40% of individuals who suffer an initial ankle sprain go on to develop CAI [8]. This condition is characterised by repeated episodes of ankle giving way, persistent pain, sensorimotor deficits, and impaired function, which may significantly impact athletic performance and daily activities [9,10]. Moreover, CAI is associated with long-term sequelae such as early-onset post-traumatic osteoarthritis and functional disability [11]. Traditional explanations of CAI have largely focused on mechanical insufficiencies such as ligamentous laxity and impaired proprioception; however, this perspective fails to account for the neurophysiological dimension of the condition, in which AMI plays a central role [12,13].

Following an ankle injury, mechanoreceptors within the joint capsule, ligaments, and surrounding tissues are often damaged, disrupting the normal flow of afferent input to the spinal cord and higher centres [2,14]. This altered input contributes to a cascade of inhibitory mechanisms at multiple levels of the central nervous system. At the spinal level, changes in group II, III, and IV afferents modulate interneuronal activity, leading to decreased excitability of the α-motoneuron pool [3]. In particular, circuits such as the group Ib inhibitory loop, the flexor reflex pathway, and the gamma loop have been implicated in the pathogenesis of AMI. These alterations result in the muscle’s inability to generate a full voluntary contraction, even in the absence of overt muscle damage or pain. Additionally, supraspinal contributions—including maladaptive cortical plasticity and altered descending modulation from brainstem nuclei—have been observed in chronic cases. For example, increased corticospinal excitability in the presence of reduced electromyography (EMG) amplitude suggests a compensatory effort by the central nervous system to override persistent spinal inhibition [15,16]. Such findings point to a complex, multifactorial origin of AMI, which likely includes a mixture of spinal reflex modulation, central sensitisation, and behavioural adaptations such as fear-avoidance or learned non-use [17,18].

The muscles most affected by AMI in the context of ankle instability appear to be the peroneus longus and the soleus, both of which play a key role in lateral and plantar stability of the joint during dynamic tasks such as gait, running, and landing. Electromyographic studies have shown reduced H-reflex amplitudes and altered H/M ratios in these muscles among individuals with CAI, indicating a compromised ability to activate them voluntarily [19,20]. These impairments persist beyond the acute phase and are often present even in athletes who have resumed sport-specific training, suggesting that strength training alone may not suffice to restore full neuromuscular control. The failure to address AMI can therefore lead to an incomplete or maladaptive recovery, where strength deficits and poor proprioceptive control perpetuate a vicious cycle of instability, reinjury, and further neuromuscular inhibition [2,21].

From a rehabilitation perspective, the presence of AMI poses significant challenges. Standard physiotherapy protocols that focus solely on resistance exercises or joint mobilisation may not adequately address the neural inhibition that underlies poor muscle performance. Without restoring voluntary activation, strengthening efforts may have limited effect, and the patient may remain at risk of recurrence [22,23]. Moreover, the heterogeneity of rehabilitation approaches in the literature, combined with variability in assessment methods (e.g., EMG parameters, H-reflex analysis, interpolated twitch techniques), has made it difficult to establish clear guidelines for the clinical management of AMI. Some interventions—such as manual therapy, proprioceptive training, neuromuscular electrical stimulation, and, more recently, transcranial direct current stimulation (tDCS)—have shown promising effects on AMI-related outcomes, yet their implementation remains inconsistent in clinical practice [24,25,26].

The clinical significance of AMI becomes even more pronounced when considering its implications for motor control, joint loading, and injury prevention. Muscular inhibition not only limits immediate functional recovery but may alter long-term motor strategies. Individuals with CAI often demonstrate aberrant movement patterns, including increased reliance on visual feedback, compensatory hip muscle recruitment, and impaired balance control. These adaptations may represent attempts by the central nervous system to bypass deficient ankle control, yet they can introduce new biomechanical stresses and elevate injury risk elsewhere in the kinetic chain. Therefore, a more comprehensive understanding of AMI, and how to address it effectively, is essential for advancing ankle rehabilitation beyond symptom management toward long-term functional restoration and injury resilience [6,27,28].

To date, the only quantitative synthesis focused on this topic is the meta-analysis by Kim et al. [4], in which the authors pooled data on strength, balance, and functional outcomes in patients with chronic ankle instability (CAI). While informative, their work was inherently limited to outcomes amenable to statistical aggregation and did not explore the underlying neural mechanisms—particularly the role of spinal and supraspinal inhibition. Moreover, it excluded several recent neuromodulation studies published after the search period. In contrast, the present narrative review aims to offer a more integrative and mechanistic perspective by focusing specifically on arthrogenic muscle inhibition (AMI) as a distinct neurophysiological phenomenon, and by synthesising evidence from neurophysiology, motor control, and novel rehabilitative approaches such as transcranial direct current stimulation (tDCS). This approach allows for a broader, clinically relevant understanding of how AMI contributes to CAI and how it can be targeted in rehabilitation.

Given these considerations, this narrative review aims to bridge a critical gap in the current rehabilitation literature by focusing specifically on arthrogenic muscle inhibition in the context of ankle instability and soft tissue disorders. By discussing and integrating relevant findings from the literature on its mechanisms, clinical presentation, and therapeutic approaches, this work seeks to offer a neurophysiologically informed perspective that may guide more effective treatment strategies. The ultimate objective is not only to raise awareness of AMI as a distinct clinical entity but also to critically evaluate physiotherapeutic interventions that have the potential to reduce inhibition, restore neuromuscular function, and enhance overall rehabilitation outcomes in patients with ankle joint dysfunction.

## 2. Materials and Methods

The review followed a structured yet flexible methodological framework, consistent with guidance from the SQUIRE 2.0 reporting guidelines, while remaining focused on the qualitative integration of data derived from the experimental and clinical literature [29,30].

A comprehensive literature search was conducted between April and October 2024 across five major biomedical databases: PubMed/MEDLINE, Web of Science (WOS), Scopus, Cochrane Central Register of Controlled Trials (CENTRAL), and PEDro (Physiotherapy Evidence Database). Additional sources were retrieved through Google Scholar for grey literature and reference mining, and all bibliographic records were stored, organised, and deduplicated using the Zotero reference management software. Zotero also facilitated tagging and annotation of records during full-text screening and data extraction.

The search strategy was designed to maximise both sensitivity and specificity. A combination of controlled vocabulary (e.g., MeSH terms where applicable), Boolean operators, and free-text keywords was used. The core concepts guiding the search were as follows: (1) the phenomenon of AMI; (2) ankle joint instability or dysfunction; and (3) physiotherapeutic or rehabilitative interventions. The final search strings used for each database were as follows:

- PubMed/MEDLINE: (“arthrogenic muscle inhibition”[Title/Abstract] OR “arthrogenous inhibition”[Title/Abstract] OR “neuromuscular inhibition”[Title/Abstract]) AND (“ankle instability”[MeSH Terms] OR “chronic ankle instability”[Title/Abstract] OR “functional ankle instability”[Title/Abstract]) AND (“rehabilitation”[MeSH Terms] OR “physiotherapy”[Title/Abstract] OR “manual therapy”[Title/Abstract] OR “disinhibitory modalities”[Title/Abstract]).
- Web of Science (WOS): TS = (“arthrogenic muscle inhibition” OR “arthrogenous muscle weakness” OR “reflex muscle inhibition”) AND TS = (“ankle instability” OR “chronic ankle instability”) AND TS = (“rehabilitation” OR “physical therapy” OR “manual therapy”).
- Scopus: TITLE-ABS-KEY(“arthrogenic muscle inhibition” OR “arthrogenous inhibition” OR “neurophysiological inhibition”) AND TITLE-ABS-KEY(“ankle instability” OR “ankle joint dysfunction”) AND TITLE-ABS-KEY(“physiotherapy” OR “rehabilitation” OR “manual therapy” OR “neuromuscular training”).
- Cochrane CENTRAL: (“arthrogenic muscle inhibition” OR “arthrogenous inhibition”) in Title Abstract Keyword AND (“ankle instability” OR “ankle dysfunction”) AND (“physiotherapy” OR “manual therapy” OR “rehabilitation”)
- PEDro: search term—“arthrogenic muscle inhibition”; study types— randomised controlled trials (RCTs) and systematic reviews.
- Google Scholar: free-text searches were performed using variations, such as the following: “arthrogenic muscle inhibition” AND “ankle rehabilitation”; “neurophysiological inhibition” AND “ankle instability”. Relevant hits from the first 100 results were screened manually.

The inclusion criteria were defined as follows:

- Articles published in English and available in full text.
- Human studies involving participants with ankle dysfunction (functional or chronic ankle instability) or soft tissue injury of the tibio-talar region.
- Studies that included interventions aiming to improve neuromuscular activation, voluntary contraction, or proprioceptive control.
- Designs considered: randomised controlled trials (RCTs), systematic reviews, and meta-analyses.
- Outcomes needed to include at least one of the following: neurophysiological measures (e.g., H-reflex, H/M ratio, EMG amplitude), functional motor recovery, pain, balance, or subjective disability (e.g., FAAM, CAIT).

The exclusion criteria were as follows:

The exclusion criteria mirrored the inverse of the inclusion criteria, excluding studies unrelated to ankle AMI, with no neurophysiological outcome, or lacking a rehabilitation component.

The initial search yielded 75 articles, of which 52 were excluded due to irrelevance following title and abstract screening. A total of 23 articles were retained for full-text review. Following full-text assessment, an additional 12 studies were excluded for not meeting inclusion criteria (e.g., poor study design, non-targeted outcomes, duplicate data). In the end, 5 studies were included in the final synthesis. Five randomised controlled trials were included in this review, each targeting AMI in chronic ankle instability with different intervention strategies. Detailed characteristics of these studies, including participant demographics, intervention protocols, and main outcomes, are summarised in Table 1.

The selection process was performed independently by the reviewer. Zotero was used not only for citation management but also to tag reasons for exclusion, group articles by relevance, and extract metadata for synthesis. No formal risk-of-bias tool was applied due to the narrative nature of this review.

Data extracted from each included study comprised the following:

- Population characteristics (sample size, age, sex, and physical activity).
- Intervention types (e.g., taping, proprioceptive training, manual therapy, destabilisation devices, and tDCS).
- Outcome measures (e.g., h/M ratio, EMG activity, strength testing, balance tests, and subjective scales).
- Findings regarding effectiveness in reducing AMI or improving related functional outcomes.
- Methodological quality indicators (blinding, randomisation, control conditions, and duration of follow-up).

Instead, a narrative synthesis approach was adopted to highlight patterns, effectiveness trends, and mechanisms of action, with a particular focus on clinical translatability.

The review aimed to maintain clinical relevance by interpreting results not only through statistical significance, but also through physiological plausibility and applicability to physiotherapy practice, emphasising interventions that could be implemented in daily rehabilitation routines. This includes a critical reflection on how each intervention might modulate central or peripheral mechanisms of AMI, and whether such effects can be maintained over time or generalised across populations.

## 3. Results

Among the five included studies [18,23,25,26,31], two [18,23] directly evaluated changes in neural excitability, whereas the remaining three [25,26,31] focused exclusively on functional outcomes such as strength, balance, or proprioception.

### 3.1. Spinal and Cortical Excitability

Among the five included studies, two directly evaluated changes in neural excitability, distinguishing between spinal and supraspinal effects. Chou et al. [18] explored the acute response of segmental spinal circuits following the application of fibular reposition taping (FRT). It was hypothesised that this intervention would restore fibular alignment and influence joint mechanoreceptors, thereby modulating afferent feedback. In a small cohort of 12 physically active participants with chronic CAI, the application of tensioned tape resulted in a statistically significant increase in the soleus H-reflex–M-wave (H/M) ratio, from a baseline mean of 0.42 to 0.61 (*p* < 0.05). This reflects a disinhibition of spinal excitability, suggesting that FRT can acutely modulate the monosynaptic reflex pathway. Notably, no significant differences were observed in the peroneus longus H/M or in V/M ratios (volitional drive), indicating that the effect was both muscle-specific and predominantly spinal. These findings, while promising, must be interpreted with caution due to the limited sample size and lack of longitudinal follow-up.

Conversely, Bruce et al. [26] employed non-invasive brain stimulation—specifically anodal tDCS over the primary motor cortex (M1)—in conjunction with eccentric exercise. In their double-blind randomised controlled trial (*n* = 26), significant reductions in resting motor threshold (RMT) and cortical silent period (CSP) were observed post-intervention. RMT declined from 43.6% to 38.2% of maximal stimulator output (*p* = 0.01), while CSP duration was reduced by an average of 16.7 milliseconds (*p* < 0.05). These findings denote an upregulation of corticospinal excitability and a downregulation of intracortical inhibition—two neurophysiological processes that are central to reversing the central components of AMI. The enhanced excitability was also accompanied by an increase in EMG amplitude of the peroneus longus, providing peripheral corroboration of the central effect. This study is among the first to demonstrate a direct cortical mechanism contributing to functional improvement in CAI, supporting the use of neuromodulation techniques in rehabilitation settings.

### 3.2. Pain Reduction and Sensory Modulation

Pain modulation was assessed primarily through self-reported pain intensity and pressure pain thresholds. Plaza-Manzano et al. [23] provided compelling evidence for the efficacy of combining manual therapy with proprioceptive and strengthening exercises. Over a four-week intervention, participants in the manual therapy group reported a dramatic decrease in pain scores, as measured by the Visual Analogue Scale (VAS), dropping from 5.2 ± 1.1 to 0.8 ± 0.5 (*p* < 0.001). Additionally, pressure pain thresholds (PPT) recorded at the anterior talofibular ligament increased by 48% from baseline, suggesting the desensitisation of local nociceptive input. These sensory gains may reflect the activation of segmental inhibitory circuits, modulation of dorsal horn neurons, or descending supraspinal mechanisms. Notably, such changes were not observed in the exercise-only group, highlighting the added value of joint mobilisation and neurodynamic techniques in pain control. Importantly, none of the other four studies explicitly assessed pain as a primary outcome, indicating a potential gap in outcome standardisation in this field.

### 3.3. Muscle Strength and Electromyographic Activation

Three studies evaluated the effects of intervention on isometric strength and/or muscle activation via surface EMG. Plaza-Manzano et al. [23] demonstrated clinically meaningful gains in both plantarflexor and dorsiflexor strength after their intervention. In the manual therapy group, plantarflexor strength improved by 26% and dorsiflexor strength by 21%, compared to 14% and 11%, respectively, in the exercise-only group. These increases were accompanied by improved joint mobility and functional scores, supporting the hypothesis that manual therapy may facilitate neuromuscular activation by alleviating joint restriction and altering afferent input.

In the study by Bruce et al. [26], the EMG amplitude (RMS) of the peroneus longus increased significantly in the active tDCS group—from a pre-intervention value of 115 μV to 184 μV post-intervention (*p* < 0.05)—suggesting enhanced voluntary muscle recruitment. This change was not observed in the sham stimulation group. These results reinforce the emerging concept that cortical disinhibition is a modifiable contributor to chronic muscle underactivation in CAI. Donovan et al. [31], on the other hand, recorded mild increases in peroneal muscle activity following rehabilitation with destabilising devices, although these changes did not reach statistical significance. While their findings suggest that sensorimotor challenge may engage stabilising musculature more effectively, the absence of EMG normalisation suggests that structural rehabilitation may not be sufficient to fully reverse underlying AMI.

### 3.4. Dynamic Balance and Motor Control

All studies that included functional assessments reported improvements in dynamic balance, albeit to varying extents. Donovan et al. [31] used the Star Excursion Balance Test (SEBT) and found that participants in both groups improved reach distance by 8–12% across multiple directions. Interestingly, the group using destabilising footwear (a pivot-heel boot or sandal) did not outperform the standard exercise group in a statistically significant way. This calls into question the additional value of unstable devices in rehabilitation and suggests that proprioceptive improvement may plateau after a certain stimulus threshold is reached.

Bruce et al. [26] also assessed dynamic motor control using single-leg hop distance and medio–lateral sway indices. The tDCS group improved in both parameters, while the sham group did not. Single-leg hop distance increased by an average of 14 cm (*p* < 0.01), and sway reduced by 18% (*p* = 0.03). These data suggest that supraspinal stimulation not only facilitates motor unit recruitment but also contributes to complex coordination during dynamic tasks, which are essential for injury prevention and return to sport.

Sierra-Guzmán et al. [25] took a different approach by measuring **muscle reaction time**, which reflects the latency between a perturbation and the onset of muscle activity—a critical marker of neuromuscular readiness. In their six-week training protocol using BOSU platforms with and without whole-body vibration (WBV), they found significant improvements in reaction time for the peroneus longus, peroneus brevis, and tibialis anterior only in the WBV group. For example, peroneus longus onset latency was reduced from 96 ms to 72 ms (*p* < 0.05), an effect maintained at six-week follow-up. This underscores the role of rapid reflex pathways in ankle protection and adds nuance to strength-focused paradigms, which may neglect reactive neuromotor timing.

### 3.5. Subjective Function and Perceived Stability

Subjective function, as measured by the Cumberland Ankle Instability Tool (CAIT) or the Foot and Ankle Ability Measure (FAAM), consistently improved across studies. Plaza-Manzano et al. [23] reported an increase in CAIT score from 16.4 ± 3.2 to 29.0 ± 1.4, reaching levels indicative of restored functional integrity. Similarly, Bruce et al. [26] documented CAIT gains from 17.8 to 26.4 post-intervention with tDCS. Donovan et al. [31], though not using CAIT, recorded FAAM improvements of 16–19%, demonstrating that subjective perceptions of stability and function are sensitive to therapeutic change. These measures are important not only as proxies for physical recovery, but also because they correlate with return-to-play criteria and risk of reinjury. Improvements in these scores suggest not only neuromuscular rehabilitation but also increased patient confidence, which is essential for long-term adherence and athletic performance.

A summarised overview of the main neurophysiological and functional outcomes across all five studies is presented in Table 2, highlighting key improvements and intervention-specific effects.

## 4. Discussion

The findings of this narrative review offer a multifaceted perspective on the neuromechanical and clinical responses to targeted interventions aimed at mitigating arthrogenic muscle inhibition (AMI) in individuals with chronic ankle instability (CAI). Despite the relatively small number of high-quality studies available, the synthesis reveals a pattern of outcome-specific efficacy, suggesting that AMI in CAI is a complex, multilevel phenomenon that requires equally multilevel therapeutic strategies.

One of the most compelling findings is the differential impact of interventions on spinal versus cortical excitability. Chou et al.’s [18] study on fibular reposition taping (FRT) demonstrates a selective and short-term facilitation of the soleus H-reflex, a hallmark indicator of segmental excitability. This reinforces the notion that peripheral mechanical interventions may modulate reflex pathways through altered afferent feedback, possibly by unloading joint mechanoreceptors or reducing aberrant proprioceptive input [32,33]. However, the lack of effect on other muscles and the absence of change in volitional drive (V/M ratio) suggest that spinal-level modulation alone may be insufficient for comprehensive neuromuscular restoration. This is consistent with prior research in the knee and hip, where joint mobilisation alone has shown transient reflex benefits but limited effects on voluntary activation or functional carryover [5].

More promising, in terms of long-term motor recovery, are the results reported by Bruce et al. [26], who applied anodal tDCS over the primary motor cortex (M1). Their study demonstrated a significant reduction in resting motor threshold and cortical silent period, indicative of enhanced corticospinal excitability and reduced intracortical inhibition. These neurophysiological changes were paralleled by increased EMG amplitude, improved dynamic balance, and higher CAIT scores [34]. The fact that tDCS led to simultaneous improvements in both physiological markers and clinical outcomes suggests that central disinhibition may be a critical, yet underexploited, therapeutic target in CAI rehabilitation [35]. While the sample size was modest and the follow-up period short, the coherence between cortical metrics and functional improvement supports the biological plausibility of these findings.

From a sensory perspective, the strong analgesic effect observed in Plaza-Manzano et al.’s [23] trial adds weight to the role of manual therapy not just as a biomechanical correction technique, but as a neuromodulatory intervention. The marked reduction in pain intensity and increase in pressure pain threshold indicate that joint mobilisation may engage both segmental and descending inhibitory systems, reducing nociceptive gain and facilitating motor output [36,37]. Pain and AMI are intricately linked via spinal reflex arcs and supraspinal processing; thus, reducing pain may indirectly restore motor function by disinhibiting the motor cortex and spinal circuitry. Interestingly, this study also showed superior improvements in strength and range of motion compared to exercise alone, reinforcing the concept that pain modulation may be a necessary precondition for effective muscle activation.

Nonetheless, the influence of strength and sensorimotor training should not be understated. In both the studies by Plaza-Manzano et al. [23] and Donovan et al. [31], proprioceptive and resistance exercises yielded improvements in function, albeit to differing extents. While Plaza-Manzano et al. [23] reported a marked increase in plantarflexor and dorsiflexor strength, Donovan et al.’s [31] data showed only mild gains, particularly when using destabilising devices. This raises important questions about the marginal utility of additional proprioceptive challenges in already functional populations and the dose–response relationship between sensory stimulation and functional gain. The absence of significant EMG changes in Donovan et al.’s [31] trial further implies that peripheral adaptations may lag behind central recovery, or that their training duration was insufficient to elicit measurable neurophysiological effects.

The study by Sierra-Guzmán et al. [25] uniquely highlights the value of assessing reaction time as a clinical outcome. Often overlooked, reaction time serves as a proxy for neuromuscular readiness and real-time joint protection. Their findings—that whole-body vibration (WBV) training significantly reduced latency in the peroneus longus, brevis, and tibialis anterior—underscore the potential for frequency-based stimuli to entrain spinal reflexes and enhance protective muscular responses [38,39]. However, the lack of corresponding torque or strength gains suggests that WBV may improve reactivity without enhancing contractile capacity. This delineation is clinically relevant: in return-to-sport decision making, quick activation may be as important as absolute force production, especially in preventing reinjury.

While all studies reported improvements in subjective stability, the instruments used varied, ranging from the CAIT to the FAAM, which limits direct comparisons. Nevertheless, improvements across these scales reflect not only biomechanical restoration but also enhanced psychological confidence in the joint—an often-neglected component of rehabilitation. Given the known relationship between kinesiophobia and reinjury, the observed improvements in perceived stability may be critical in reducing recurrence rates, though none of the studies included long-term follow-up data to support this assertion.

While some results appear promising, the limitations inherent to narrative reviews must be acknowledged, particularly the absence of quantitative synthesis and structured quality appraisal, this review also highlights several important limitations within the current body of literature. Firstly, sample sizes across studies were uniformly small (ranging from 12 to 56 participants), limiting statistical power and generalisability. Secondly, outcome heterogeneity remains a significant challenge. While some studies focused on neurophysiological metrics such as H/M ratio, CSP, and EMG, others prioritised subjective or functional outcomes. This variability makes synthesis difficult and underscores the need for standardised outcome sets in AMI research. Furthermore, intervention durations ranged from a single session to six weeks, making it difficult to determine optimal treatment length or frequency. The lack of follow-up assessments in most studies further limits understanding of long-term efficacy and sustainability of gains.

Another critical gap is the underrepresentation of female participants in certain trials, despite the known sex differences in joint laxity, neuromuscular control, and injury risk. Additionally, most interventions targeted young, physically active adults, with little evidence available for older adults or those with comorbidities. Future studies should strive to include more diverse populations to enhance external validity.

Lastly, methodological transparency remains suboptimal in several studies. Blinding procedures were inconsistently reported, and few studies employed sham conditions or intention-to-treat analyses. The use of advanced technologies such as tDCS or WBV also raises questions about cost-effectiveness and accessibility in routine clinical practice, which should be addressed in implementation studies [40,41,42].

In sum, the available evidence suggests that AMI in CAI can be addressed through a combination of interventions targeting spinal, supraspinal, and peripheral mechanisms. Manual therapy and sensorimotor training remain foundational, but neuromodulation techniques like tDCS show promising early results [18,25,31]. A multimodal approach tailored to individual neurophysiological profiles may offer the best chance of restoring full function and preventing reinjury. However, more rigorous, adequately powered trials with standardised outcomes and longer follow-up periods are urgently needed to confirm these preliminary findings and inform clinical guidelines.

### Clinical Practice Implications

This review reinforces the importance of adopting a multimodal and neurophysiologically informed approach to the rehabilitation of patients with chronic ankle instability (CAI), particularly in the presence of arthrogenic muscle inhibition (AMI). Interventions that combine peripheral, spinal, and cortical strategies may provide superior clinical outcomes compared to isolated modalities [43,44].

Manual therapy, when integrated with proprioceptive and strengthening exercises, is effective in reducing pain, enhancing pressure pain thresholds, and restoring joint mobility—creating a more favourable environment for motor reactivation. These effects are particularly relevant in the early stages of rehabilitation, where pain-related inhibition may compromise muscle performance and neuromuscular retraining [45].

Spinal-level interventions such as fibular reposition taping can provide transient reflex disinhibition, offering an adjunctive strategy for sessions focused on sensorimotor re-education. However, their short-term effects highlight the necessity for sustained and progressive loading strategies to consolidate neuromotor gains.

Cortical modulation using transcranial direct current stimulation (tDCS) is emerging as a promising tool to enhance corticospinal excitability and voluntary muscle recruitment. Though not yet widely adopted in standard physiotherapy settings, the integration of tDCS with eccentric loading may be beneficial for selected patients exhibiting persistent central inhibition.

Clinicians should also consider incorporating balance and reaction-time training—especially using perturbation-based or vibration platforms—to address deficits in neuromuscular responsiveness, which are critical for injury prevention and dynamic joint stability.

Finally, subjective functional tools such as the Cumberland Ankle Instability Tool (CAIT) [46] and the Foot and Ankle Ability Measure (FAAM) should be routinely used to monitor patient-perceived improvements and guide return-to-sport decisions. Enhancing patients’ confidence and perceived control over joint function is a key determinant of long-term adherence and reinjury prevention.

## 5. Conclusions

AMI represents a critical yet often-overlooked barrier to functional recovery in patients with CAI. Although the current evidence suggests potential benefits from neural and functional rehabilitation approaches, the lack of standardised protocols and limited study quality call for more targeted research. Clinicians should remain aware of AMI’s implications and consider neuromodulatory strategies alongside conventional therapies to optimise patient outcomes.

## Figures and Tables

**Table 1:** Summary of included studies on arthrogenic muscle inhibition in chronic ankle instability.

Study (Author, Year, Design)	Participants (Age, Sex, N)	Intervention (Methods)	Results (Numerical Outcomes)	Main Outcomes Achieved
Chou et al., 2013, RCT [18]	12 young adults (mean age 21.5 ± 2.3; 7M/5F)	Fibular reposition taping vs. sham taping; H/M and V/M ratios recorded pre/post (5 min).	↑ Soleus H/M ratio (*p* < 0.05); no change in peroneus or V/M ratio.	Short-term increase in spinal excitability (soleus); muscle-specific and transient effect.
Plaza-Manzano et al., 2018, RCT [23]	56 active adults (mean age 24.3 ± 3.8; 30M/26F)	4-week proprioceptive and strengthening exercise vs. same and manual therapy; pain, strength, ROM, CAIT, and PPT assessed.	VAS ↓ from 5.2 to 0.8; CAIT ↑ from 16.4 to 29.0; strength ↑ significantly; PPT ↑; ROM ↑.	Superior pain reduction, function, strength, and mobility when manual therapy is added.
Donovan et al., 2019, RCT [31]	26 young adults (mean age 22.7 ± 1.9; 14M/12F)	4-week rehabilitation with vs. without destabilising device; EMG, FAAM, SEBT, strength, and ROM measured.	FAAM improved in both groups; SEBT reach ↑ more with device but not statistically significant; EMG ↑ mildly.	Minor added value of destabilising device over standard rehab; possible EMG facilitation.
Sierra-Guzmán et al., 2018, RCT [25]	50 physically active adults (mean age 23.6 ± 2.1; 28M/22F)	6-week training using BOSU with or without whole-body vibration; reaction time and torque assessed.	WBV group: ↓ reaction time (PL, PB, TA); no torque changes; maintained at 6 weeks.	Improved neuromuscular responsiveness (faster reaction time) even without strength gain.
Bruce et al., 2020, Double-blind RCT [26]	26 adults with CAI (mean age 24.1 ± 3.5; 13M/13F)	10 sessions of tDCS (anodal M1) and eccentric training vs. sham; RMT, CSP, EMG, dynamic balance, and CAIT analysed.	↓ RMT and CSP in tDCS group; ↑ EMG amplitude; improved hop test and postural scores; CAIT ↑.	Central disinhibition and functional improvement via tDCS; promising but needs follow-up.

Legend: ↑ = increases/enhances; ↓ = reduces/lowers; BOSU = both sides up (unstable training platform); CAI = chronic ankle instability; CAIT = Cumberland Ankle Instability Tool; CSP = cortical silent period; EMG = electromyography; FAAM = Foot and Ankle Ability Measure; FRT = fibular reposition taping; H/M ratio = Hoffmann Reflex to M-wave Ratio; M1 = primary motor cortex; PB = peroneus brevis; PL = peroneus longus; PPT = pressure pain threshold; RCT = randomised controlled trial; RMT = resting motor threshold; ROM = range of motion; SEBT = Star Excursion Balance Test; TA = tibialis anterior; tDCS = transcranial direct current stimulation; VAS = Visual Analogue Scale; V/M ratio = Voluntary Motor Drive to M-wave Ratio; WBV = whole-body vibration.

**Table 2:** Summary of main outcomes across studies (↑ = improvement, ↓ = reduction, – = not assessed).

Study	Spinal Excitability	Cortical Excitability	Pain Reduction	Strength	Balance/Motor Control	Subjective Stability
Chou et al., 2020 [18]	↑ Soleus H/M	–	–	–	–	–
Plaza-Manzano et al., 2018 [23]	–	–	↓↓ VAS, ↑ PPT	↑↑ PF/DF strength	↑ CAIT	↑↑ CAIT
Donovan et al., 2019 [31]	–	–	–	↗	↑ SEBT	↑ FAAM
Sierra-Guzmán et al., 2018 [25]	–	–	–	–	↓ Reaction time	–
Bruce et al., 2020 [26]	–	↑ RMT ↓ CSP	–	↑ EMG	↑ Hop/↓ Sway	↑ CAIT

## Data Availability

No new data were created or analysed in this study.
